# Bifurcation of neural firing patterns driven by potassium dynamics and neuron–electrode geometry during high-frequency stimulation

**DOI:** 10.1371/journal.pcbi.1014228

**Published:** 2026-04-28

**Authors:** Yue Yuan, Junyang Zhang, Chen Wang, Hao Yan, Ning Zhang, Kun Zhang, Zheshan Guo, Zhaoxiang Wang

**Affiliations:** 1 Research Center for Life Sciences Computing, Zhejiang Lab, Hangzhou, China; 2 State Key Laboratory of Digital Medical Engineering, Key Laboratory of Biomedical Engineering of Hainan Province, Sanya Research Institute of Hainan University, School of Biomedical Engineering, Hainan University, Sanya, China; 3 Key Lab of Biomedical Engineering for Ministry of Education, College of Biomedical Engineering and Instrument Science, Zhejiang University, Hangzhou, China; University of Edinburgh, UNITED KINGDOM OF GREAT BRITAIN AND NORTHERN IRELAND

## Abstract

High-frequency stimulation (HFS), the basis of deep brain stimulation, elicits diverse neuronal responses, yet the mechanisms remain unclear. Classical conduction block theories cite sodium channel inactivation and axonal failure but cannot explain the abrupt, reproducible firing transitions observed *in vivo*. Here, we combine single-unit recordings from rat CA1 neurons with a biophysically detailed multi-compartment model to examine how HFS shapes axonal excitability. The results show that neuronal responses are governed by two coupled factors: the electrode–axon geometry and peri-axonal extracellular potassium ([K⁺]_o_) dynamics. Small changes in either parameter reliably triggered bifurcation-like transitions between tonic, clustered, and low-rate regular firing. Conduction block preceded initiation failure with increasing electrode-axon distance, whereas elevated [K⁺]_o_ shifted membranes between excitable and non-excitable states. This unified bifurcation framework extends the conduction block hypothesis, recasts axons as nonlinear elements, and provides mechanistic insights to optimize electrode placement, stimulation tuning, and closed-loop neuromodulation strategies.

## Author summary

Deep brain stimulation has transformed the treatment of neurological disorders, yet how high-frequency electrical pulses reshape brain activity remains a puzzle. Our work shows that neurons do not simply turn “on” or “off” under stimulation but instead switch between multiple stable states, depending on their physical surroundings and the chemistry of their environment. Using a combination of animal recordings and detailed computer modeling, we found that tiny shifts in electrode position or potassium levels around nerve fibers can push neurons into qualitatively different firing modes—steady, clustered, or silent. These rapid transitions follow the principles of nonlinear dynamics, revealing that axons behave like complex systems capable of bifurcation rather than passive wires transmitting signals. This new perspective helps explain why identical stimulation can yield opposite outcomes across brain regions or patients. By recognizing and harnessing these nonlinear mechanisms, future neurostimulation therapies may be tuned with greater precision to guide brain circuits toward beneficial activity patterns while minimizing side effects.

## 1. Introduction

High-frequency stimulation (HFS), the standard paradigm of deep brain stimulation (DBS), is an established therapy for a range of neurological and psychiatric disorders, including Parkinson’s disease, essential tremor, epilepsy, and depression [1–3]. Despite its clinical efficacy, the underlying mechanisms are not fully understood [4,5].

Early parallels between DBS and surgical lesions in Parkinson’s disease fostered the view that HFS primarily suppresses local neuronal activity [5,6]. Subsequent studies, however, demonstrated increased downstream activity mediated by the activation of nearby axons [2,7]. This coexistence of local inhibition and distal excitation has shifted the field away from a binary “inhibition versus excitation” framework toward a dynamical view, in which HFS modulates firing patterns rather than simply silencing or activating neurons [5,7]. Both experimental and computational studies have shown that periodic HFS can induce diverse neuronal responses, including tonic, intermittent regular, and clustered firing [8–13]. This raises a critical unresolved question: how can uniform HFS pulses generate such heterogeneous firing patterns?

Axons are preferentially recruited by extracellular pulse stimulation due to their abundance and low rheobase current [14,15]. Sustained HFS evokes repetitive action potentials that enhance potassium efflux, elevating peri-axonal extracellular potassium concentration ([K⁺]_o_) and inducing depolarization block [16,17]. However, depolarization block is transient; axons can repolarize and recover their ability to fire [5,11,18]. These observations led to the “partial conduction block” hypothesis, which attributes firing diversity to variable conduction failures along axons [8,10–12]. However, axonal initiation and propagation are governed by nonlinear dynamics rather than a binary switch between conduction and block. Three key points remain insufficiently addressed: (1) how does neuron–electrode geometry influence axonal excitability? (2) how do peri-axonal [K⁺]_o_ dynamics modulate neuronal firing patterns? and (3) how do bifurcations in firing patterns emerge near critical thresholds of excitability?

In this study, we combine *in vivo* recordings from rat hippocampal CA1 neurons with a biophysically detailed multi-compartment model to address these questions. The results show that axonal excitability is jointly governed by electrode–neuron geometry and peri-axonal [K⁺]_o_ dynamics. Small perturbations in either parameter drive bifurcation-like transitions among tonic, clustered, and intermittent firing, even under identical stimulation. These results establish axons as nonlinear computational elements whose responses arise from the interplay of local ionic dynamics and external electric fields.

## 2. Materials and methods

### 2.1. Animal experiments

#### 2.1.1 Ethics statement.

All animal experimental procedures were approved by the Animal Ethics Committee of Hainan University (No. HNUAUCC-2025–00430).

#### 2.1.2 Animal surgery, stimulating and recording.

Fourteen adult male Sprague–Dawley rats (250–400 g) were used. Animals were anesthetized with urethane (1.25 g/kg, i.p.) and positioned in a stereotaxic apparatus (Stoelting Co., USA). Surgical procedures followed previously established protocols [10,19].

Briefly, a 16-channel recording electrode array (RE; #A1 × 16-Poly2–5mm-50s-177, NeuroNexus Technologies, USA) was implanted into the left hippocampal CA1 region (anterior–posterior: -3.5 mm; medial–lateral: 2.7 mm; dorsal–ventral: ~ 2.5 mm) to collect extracellular potentials. A concentric bipolar stimulation electrode (SE; #CBCSG75, FHC Inc., USA) was inserted into the alveus (anterior–posterior: -4.8 mm; medial–lateral: 2.7 mm; dorsal–ventral: ~ 1.7 mm) to stimulate CA1 pyramidal axons. Stimulation of alveus fibers evoked antidromic activation of pyramidal soma near the recording sites. Electrode placements were verified by analyzing both single-unit activity (SUA) and evoked potential waveforms across the 16 recording channels.

Stimulation consisting of biphasic constant-current pulses (negative phase followed by positive phase, 0.1 ms per phase) was generated by a programmable stimulator (Model 3800, A-M Systems Inc., USA). To ensure reliable SUA recordings while avoiding activation of large neuronal populations, stimulation current was adjusted within 0.03–0.1 mA. The stimulation frequency was 100 Hz, and trains lasted 1 min.

Extracellular potentials collected by the RE were amplified 100-fold using a 16-channel amplifier (Model 3600, A-M Systems Inc., USA) with a filtering range of 0.3–5000 Hz. Signals were then digitized at 20 kHz using a PowerLab acquisition system (C Series, ADInstruments Pty Ltd., Australia) and stored for offline analysis.

### 2.2. Simulation models

We developed a computational model of a CA1 pyramidal neuron to investigate how biophysical parameters influence firing patterns. The model was adapted from our previous studies and extended to incorporate mechanisms of extracellular potassium accumulation and clearance [16,20,21]. All simulations were performed in the NEURON environment (version 8.2) [22].

#### 2.2.1. Morphological and electrical properties.

**Fig 1:**
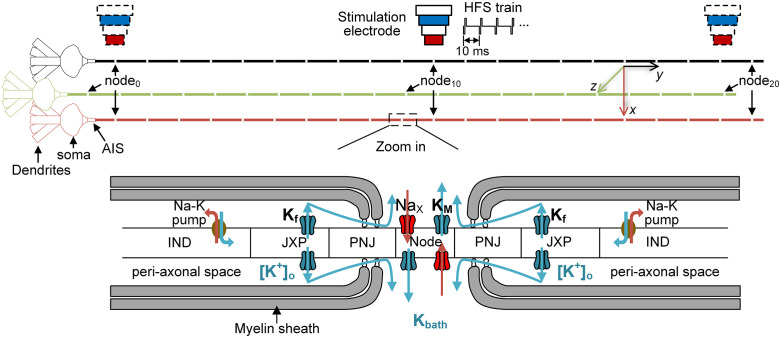
Schematic of the simulated CA1 pyramidal neuron model. *Top*: Overall morphology of the model, consisting of a soma, five dendritic branches, an axonal initial segment (AIS), and a long myelinated axon containing 21 nodes of Ranvier. A stimulation electrode (SE) was positioned above the axon, typically over node_10_ but also adjusted to other nodes when required (SE shown with dashed outlines). Neurons in different colors illustrate varying distances from the SE, with red indicating displacement along the *x*-axis and green along the *z*-axis. *Bottom*: Enlarged view of a nodal region and adjacent internodal sections, including the paranodal junction (PNJ), juxtaparanode (JXP), and internode (IND). Voltage-gated sodium channels (Na_X_) and slow M-type potassium channels (K_M_) are concentrated at the node, whereas fast potassium channels (K_f_) are distributed along the JXP. Blue arrows denote K⁺ efflux through potassium channels, axial and radial diffusion from the peri-axonal space to the extracellular environment, and active reuptake by Na–K pumps. Red arrows indicate Na⁺ flux.

The model included a soma, five dendritic branches, an axonal initial segment (AIS), and a long myelinated axon with 21 nodes of Ranvier (node_0_ to node_20_). Each pair of adjacent nodes was separated by an internodal segment composed of two paranodal junctions (PNJ), two juxtaparanodes (JXP), and one internode (IND) (Fig 1). Morphological parameters are summarized in Table 1.

**Table 1:** Morphological parameters of the CA1 pyramidal model.

Parameter	Value
**Soma and dendrites**	
Soma length and maximum diameter	15 and 10 μm
AIS length and diameter	50 and 1 μm
Dendrite length and diameter (each)	20 and 2.5 μm
**Myelinated axon**	
Axon diameter	1 μm
Internode segment length	100 μm
Node length and diameter	1 and 0.7 μm
PNJ length and diameter	3 and 0.7 μm
JXP length and diameter	5 and 0.8 μm
IND length and diameter	83 and 0.8 μm
Peri-axonal space width outside PNJ	0.004 μm
Peri-axonal space width outside JXP and IND	0.012 μm

AIS: axonal initial segment; PNJ: paranodal junction; JXP: juxtaparanode; IND: internode.

Membrane dynamics were based on the Hodgkin-Huxley formalism [23,24]. Passive and active electrical properties were adapted from our prior work and are summarized in Table 2. We made several modifications to the parameters within their physiological range to better match the experimental results. Specifically, the maximum conductance of voltage-gated sodium channels (Na_x_) and slow M-type potassium channels (K_M_) at the nodes were set to 0.86 S/cm² and 0.02 S/cm², respectively, while the maximum conductance of fast potassium channels (K_f_) at the JXP was set to 0.02 S/cm² [24,25]. The spatial distribution of these channels across nodes and adjacent internodal regions is illustrated in Fig 1.

**Table 2:** Electrical parameters of the CA1 pyramidal model.

Parameter	Value
**General**	
Membrane capacitance	1 μF/cm^2^
Leakage reversal potential	-65 mV
Na^+^ Nernst potential	45 mV
K^+^ Nernst potential	-90 mV
Ca^2 +^ Nernst potential	140 mV
**Soma and dendrites**	
Maximum voltage-gated Na^+^ conductance	8 × 10^–3^ S/cm^2^
Maximum slow non-inactivating K^+^ conductance	3 × 10^–5^ S/cm^2^
Maximum fast voltage-gated K^+^ conductance	2 × 10^–3^ S/cm^2^
Maximum Ca^2 +^ -dependent K^+^ conductance	3 × 10^–4^ S/cm^2^
Maximum high-voltage activated Ca^2 +^ conductance	3 × 10^–5^ S/cm^2^
**Axonal initial segment**	
Maximum fast voltage-gated Na^+^ conductance	0.11–0.32 S/cm^2^
Maximum fast voltage-gated K^+^ conductance	0.02–0.1 S/cm^2^
**Axonal node**	
Maximum voltage-gated Na^+^ conductance	0.86 S/cm^2^
Maximum slow M-type voltage-gated K^+^ conductance	0.02 S/cm^2^
**Internode section**	
Maximum fast voltage-gated K^+^ conductance on JXP	0.02 S/cm^2^
Myelin conductance	0.001 S/cm^2^
Myelin capacitance	0.1 μF/cm^2^

The SE was positioned above the axon, typically at the central node (node_10_), but varied across different nodes depending on the experimental design. The extracellular electric field generated by the SE was simulated using COMSOL Multiphysics 5.3 (COMSOL Inc., Sweden). Both the electrode parameters (geometry and material) and stimulation parameters (biphasic current pulses) used in the COMSOL simulation were consistent with the experimental setup (see S1 File). Electric fields at multiple spatial locations were exported and incorporated into NEURON to examine how electrode–axon and electrode–soma distances influenced firing behavior.

#### 2.2.2 Potassium accumulation and clearance in the peri-axonal space.

To model how [K⁺]_o_ dynamics influence neuronal firing patterns, mechanisms of potassium accumulation and clearance were incorporated into the peri-axonal space [16,20,21].

At baseline, [K⁺]_o_ in the peri-axonal space was set equal to the potassium concentration in the surrounding bath solution (K_bath_). During sustained stimulation, outward K⁺ currents through K_f_ channels at the JXP membrane led to potassium accumulation in the narrow peri-axonal space, elevating [K⁺]_o_ while K_bath_ remained constant. In subsequent simulations, the initial [K⁺]_o_ was varied by adjusting K_bath_.

Accumulated K ⁺  was cleared through two mechanisms: (1) axial and radial diffusion from the peri-axonal space into the extracellular space surrounding the nodes, and (2) reuptake via Na-K pumps uniformly distributed along the internodal membrane (blue arrows in Fig 1). Both axial and radial diffusion followed Fick’s law:


$$J=D\times A\times \frac{d[{\text{K}}^{+}{]}_{\text{o}}}{dx}$$

where *J* is the diffusion flux, *D* is the diffusion coefficient, *A* is the cross-sectional area of the diffusion zone, [K⁺]_o_ is the potassium concentration in the peri-axonal space, and *x* is the diffusion distance [16,20,21].

Potassium clearance by the Na–K pump, which imports two K⁺ ions and exports three Na⁺ ions per ATP hydrolyzed, was modeled as follows:


$$I_{\text{NaK}}=I_{\text{NaK}max}\left(\frac{[{\text{K}}^{+}{]}_{\text{o}}}{[{\text{K}}^{+}{]}_{\text{o}}+\text{KmK}}\right)\left(\frac{[{\text{Na}}^{+}\overset{\text{1.5}}{\underset{\text{i}}{]}}}{[{\text{Na}}^{+}\overset{\text{1.5}}{\underset{\text{i}}{]}}+{\text{KmNa}}^{1.5}}\right)\left(\frac{V+150}{V+200}\right)$$

where *I*_NaKmax_ is the maximum transport current per unit area, KmK and KmNa are the equilibrium binding constants for K⁺ and Na ⁺ , respectively, [Na⁺]ᵢ is the intracellular sodium concentration, and *V* is the membrane potential [16,20,21]. Parameter values are provided in Table 3.

**Table 3:** Parameters for modeling the K^+^ dynamics.

Parameter	Value
Intracellular K^+^ concentration	106 mM
Diffusion coefficient	1.85 μm^2^/ms
Cross section area of axial diffusion at PNJ	0.009 μm^2^
Cross section area of axial diffusion at JXP and IND	0.03 μm^2^
Cross section area of radial diffusion at node	8.16 μm^2^
Maximum transport current per unit area (*I*_NaKmax_)	2.46 μA/cm^2^
Equilibrium binding constant of K^+^ (KmK)	5.3 mM
Equilibrium binding constant of Na^+^ (KmNa)	27.9 mM
Intracellular Na^+^ concentration	10 mM

### 2.3. Evaluation of neuronal firing activities

#### 2.3.1 Analysis of experimental signals.

To obtain SUA of individual neurons, we performed several signal-processing steps as described in our previous work [10]. First, stimulation artifacts were removed from the raw recordings by replacing the artifact segments with interpolation lines [26]. Then, the signals were high-pass filtered (>500 Hz) to generate multiple-unit activity (MUA). Next, single-unit spikes were extracted from the MUA signals recorded from four adjacent channels in the pyramidal layer. The detailed procedure of spike detection and sorting has been previously reported [11]. The spike train of each neuron and its corresponding inter-spike interval (ISI) sequence were then obtained. Auto-correlation histograms of neurons were used to classify firing patterns: a sharp peak at short ISIs (e.g., < 10 ms) indicated burst firing, whereas a uniform distribution indicated regular firing.

To differentiate firing patterns induced by sustained HFS, we quantified the burst index (BI) and the coefficient of variance (CV) of ISI sequence. The BI was calculated by dividing the mean ISI by the mode ISI, and the CV of ISI by dividing the standard deviation of the ISI by the mean ISI. Higher BI or CV values indicate irregular or clustered firing [27,28]. Consistent with our previous study, we set the threshold for irregular clustered firing at CV > 1.5 or BI > 5.0 based on visual inspection. Then neurons with CV ≤ 1.5 and BI ≤ 5.0 were reckoned as regular firing. In contrast, neurons with CV ≤ 1.5 but BI > 5.0 were categorized as short clustered firing, and with CV > 1.5 classified as long clustered firing [10].

#### 2.3.2 Analysis of computational results.

For each simulation, a 100 Hz HFS train was delivered for 10-s using biphasic pulses (negative phase leading, phase width 0.1 ms), consistent with *in vivo* experiments. During the initial 2 s, most neurons reliably responded to each stimulus pulse with action potentials (APs). To emphasize variability across conditions, analyses focused on neuronal activity during the final 8 s of stimulation.

Our previous study showed that APs may originate from nodes adjacent to the SE under certain conditions [10], although neighboring nodes exhibit similar firing patterns. In this study, we describe AP initiation with respect to the node directly beneath the electrode for clarity, while the conclusions are not dependent on the exact initiation site. Firing patterns of simulated neurons were quantified using the following indices. AP initiation ratio: number of APs initiated at the stimulated node divided by the total number of stimulus pulses, AP conduction ratio: number of APs recorded at the soma divided by the number initiated at the stimulated node, reflecting conduction reliability, and somatic firing rate: total number of APs at the soma divided by the analysis window (typically 8 s). Based on visual inspection, in simulation, the threshold for clustered firing was adjusted to CV > 0.8.

Data from animal experiments are presented as mean ± standard deviation, with “*n*” representing the number of neurons. The statistical significance of the differences among groups was determined using non-parametric Kruskal-Wallis test with post hoc Bonferroni test.

## 3. Results

### 3.1. Distinct firing patterns induced by high-frequency stimulation with uniform parameters

**Fig 2:**
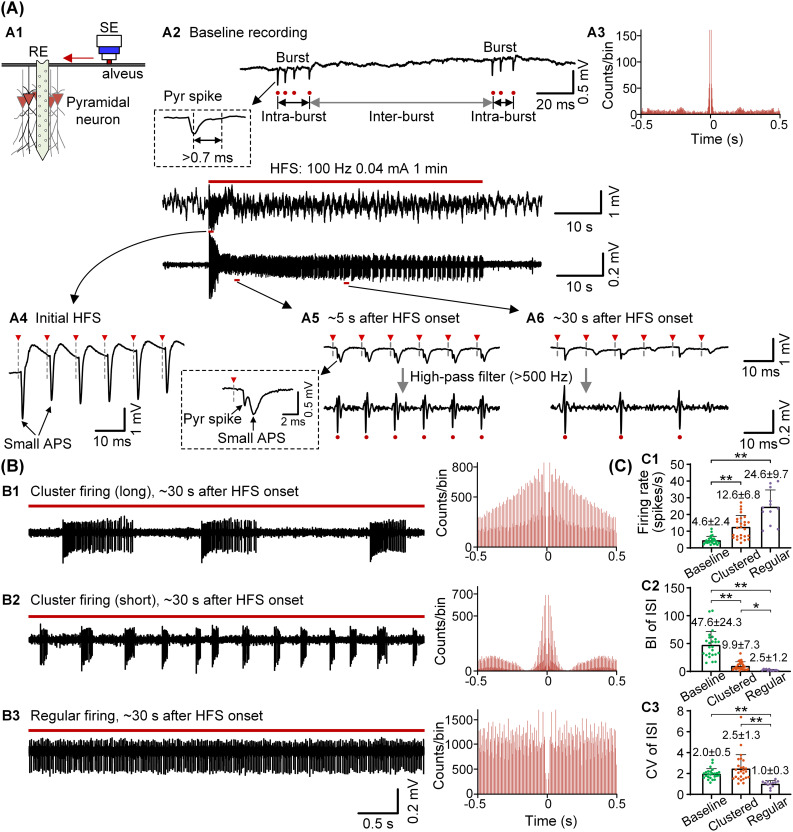
Firing of CA1 pyramidal neurons at baseline and during HFS in rat experiments. (A) (A1) Schematic showing the positions of the recording electrode (RE) and stimulation electrode (SE). (A2, A3) Example of burst firing in a pyramidal neuron during baseline recording and the corresponding auto-correlogram. (A4–A6) Representative traces showing population spike responses and subsequent tonic firing and intermittent firing during HFS. (B) Representative firing patterns during HFS: clustered firing with long clusters (B1), clustered firing with short clusters (B2), and regular firing (B3), with corresponding auto-correlograms. (C) Comparisons of mean spike firing rate (C1), BI and CV of ISI (C2 & C3) among baseline, clustered and regular firing neurons. *P < 0.05, **P < 0.01, non-parametric Kruskal-Wallis test with *post hoc* Bonferroni tests.

To examine how uniform HFS modulates neuronal firing dynamics, we applied 100 Hz trains to alveus fibers in the hippocampal CA1 region and recorded the somatic responses of pyramidal neurons (Fig 2A1). In baseline recording, the pyramidal neurons exhibited spontaneous burst firing, characterized by rapid clusters of APs with short intra-burst ISIs, producing a sharp auto-correlogram peak at ~5 ms (Fig 2A2, A3). A total of 25 pyramidal neurons were obtained during baseline recording, with the mean firing rate of 4.6 ± 2.4 spikes/s, the mean BI of 47.6 ± 24.3 and CV of ISI 2.0 ± 0.5, characterizing a typical burst firing pattern.

At the onset of HFS, each stimulus pulse reliably evoked a large-amplitude antidromic population spike, indicating synchronous activation of pyramidal neurons (Fig 2A4). Within a few seconds, the amplitude of this population spike declined due to HFS-induced axonal conduction block, allowing SUA to become clearly distinguishable. Shortly thereafter (~5 s post-onset), when conduction block remained relatively shallow, each stimulus pulse continued to evoke APs, resulting in tonic firing strictly locked to the stimulation frequency (Fig 2A5).

With continued stimulation, conduction block progressively deepened and neurons no longer followed every stimulus pulse (Fig 2A6). Despite identical stimulation parameters, heterogeneous firing patterns emerged (Fig 2B). Based on the BI and CV of ISI values for individual neurons, we classified these HFS-evoked firing responses into three categories (see Methods for details). The first group consisted of neurons with CV > 1.5, which alternated between periods of intense firing and silence, producing clustered firing with long cluster durations and long inter-cluster intervals. These patterns were characterized by slowly decaying auto-correlogram peaks (Fig 2B1). The second group included neurons with CV ≤ 1.5 and BI > 5.0, which also exhibited alternating activity but with shorter clusters and intervals, yielding more compact clusters and rapidly decaying auto-correlogram peaks (Fig 2B2). The third group neurons with CV ≤ 1.5 and BI ≤ 5.0 responded intermittently to stimulus, generating a regular firing pattern at frequencies below 100 Hz and exhibiting near-uniform auto-correlograms (Fig 2B3).

**Fig 3:**
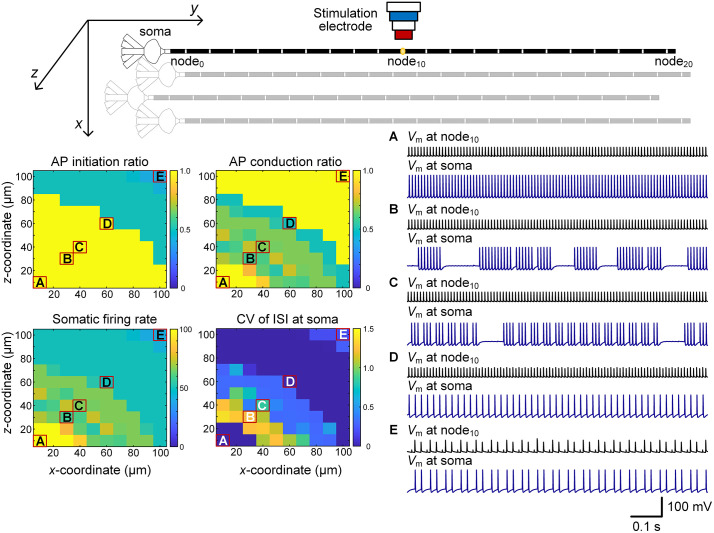
Effect of neuron–SE distance on somatic firing patterns. Representative examples of tonic firing (A), conduction failure induced short-cluster firing (B), long-cluster firing (C), regular firing (D), and initiation failure induced low-rate regular firing (E).

A total of 38 neurons were recorded from 14 rat experiments, among which 26 neurons fired in clustered pattern and 12 neurons in regular pattern. The number exceeded the 25 neurons in baseline recording, because some previously silent neurons were recruited and became active only during HFS. The mean firing rates of both clustered and regular neurons during HFS were significantly higher than those at baseline, indicating an excitatory effect of the stimulation (Fig 3C1). The BI of clustered neurons was significantly greater than the BI of regular neurons but smaller than that of baseline burst neurons (Fig 3C2). The CV of ISI for clustered neurons did not differ significantly from that of baseline burst neurons, but both were significantly higher than the CV for regular neurons, indicating the greater irregularity of clustered and burst firing (Fig 3C3).

Thus, under uniform HFS, CA1 pyramidal neurons diverged into tonic, clustered, or regular firing patterns following the initial synchronous response. This striking heterogeneity raises a fundamental question: how can seemingly identical neurons, exposed to identical stimulation, evolve into distinct firing patterns? To address this, we next used computational modeling to systematically examine how the spatial relationship between the SE and neurons modulates firing dynamics.

### 3.2. Dependence of neuronal firing patterns on neuron-electrode distance

To examine how spatial factors influence firing dynamics, we positioned the SE above the central axonal node (node_10_) and systematically varied the neuron–SE distance along the *x*- and *z*-axes (Fig 1, Fig 3). During 100 Hz HFS, both AP initiation at node_10_ and subsequent propagation to the soma were strongly distance dependent.

When the SE was positioned close to the axon (e.g., *x* = 10 µm, *z* = 10 µm), every stimulus pulse reliably initiated an AP at node_10_ (initiation ratio = 1.0), which propagated faithfully to the soma (conduction ratio = 1.0). Under these conditions, pyramidal neurons exhibited strictly stimulus-locked tonic firing at ~100 Hz, with a CV of ISI near-zero (Fig 3A). As the neuron–SE distance increased (e.g., *x* = 30 µm, *z* = 30 µm), intermittent conduction failures emerged, despite AP initiation at node_10_ remaining reliable (initiation ratio = 1.0). This selective conduction block produced clustered firing at the soma, with either long or short cluster durations, and correspondingly large ISI fluctuations (Fig 3B-3C).

With further increases in distance (e.g., *x* = 60 µm, *z* = 60 µm), conduction reliability declined more severely, such that only a fraction of initiated APs propagated to the soma. Neurons then exhibited more regular, lower-variability firing patterns (Fig 3D). At even greater distances (e.g., *x* = 100 µm, *z* = 100 µm), AP initiation itself became unreliable, with node_10_ responding only to every second or subsequent pulse. In these cases, initiated APs propagated reliably to the soma, but the overall firing rate fell below 50 Hz, yielding a low-rate regular firing pattern with ISI CV values near zero (Fig 3E).

Together, these findings show that conduction failures emerge before initiation failures as neuron–SE distance increases, driving a sequential transition from tonic to clustered, and ultimately to low-rate regular firing. Thus, even small variations in electrode placement relative to axons can critically alter neuronal firing patterns.

**Fig 4:**
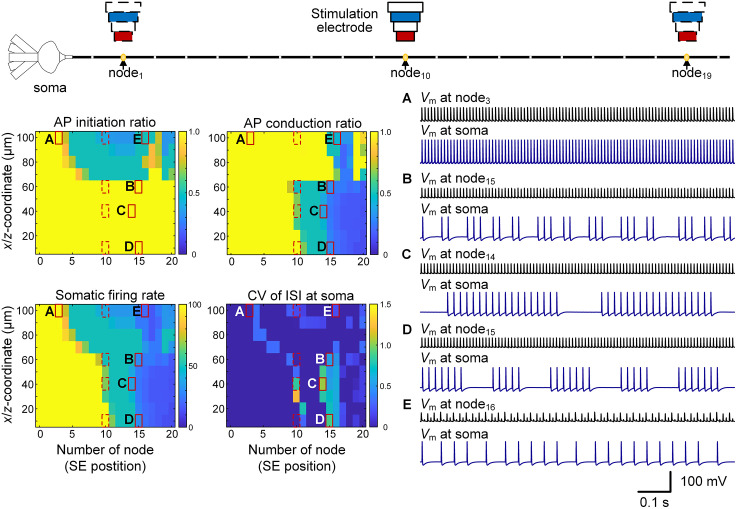
Effect of SE position along the axon on somatic firing patterns. Representative examples of tonic firing (A), clustered firing with short or long cluster durations (B–D), and low-rate regular firing (E) induced by SE displacement along the axon.

We next systematically varied SE position along different axonal nodes while keeping the neuron–SE distance constant (i.e., varying SE position along the *y*-axis). The modulatory effect of SE position depended strongly on distance. When neurons were located within ~60 µm of the SE (*x*/*z* < 60 µm), every stimulus pulse reliably initiated APs across all electrode positions. However, conduction reliability decreased as the SE was shifted distally along the axon (i.e., from node_0_ to node_20_), leading to gradual reductions in somatic firing rate (Fig 4). By contrast, when the neuron–SE distance exceeded 60 µm (*x*/*z* > 60 µm), electrode displacement primarily affected AP initiation rather than conduction. Moving the SE from proximal (node_0_) to more distal nodes (node_20_) progressively lowered AP initiation probability, with conduction failures occurring only at specific distal sites, notably around node_15_–node_18_ (Fig 4).

Tonic stimulus-locked firing was maintained only when both initiation and conduction ratios remained at 1.0 (Fig 4A). When conduction reliability declined but initiation remained intact, neurons exhibited clustered firing with either short or long cluster durations, reflected in elevated ISI CV values (Fig 4B-4D). When initiation itself became unreliable, somatic firing shifted to a low-rate, highly regular pattern with near-zero ISI CV (Fig 4E).

These results demonstrate that neuronal firing patterns depend critically on both neuron–SE distance and SE position along the axon, with conduction failures generally preceding initiation failures. Such geometric sensitivity likely contributes to the heterogeneous firing patterns observed *in vivo*. We next examined peri-axonal potassium dynamics as an intrinsic determinant of these transitions.

### 3.3. Dependence of neuronal firing patterns on potassium concentration in the peri-axonal space

**Fig 5:**
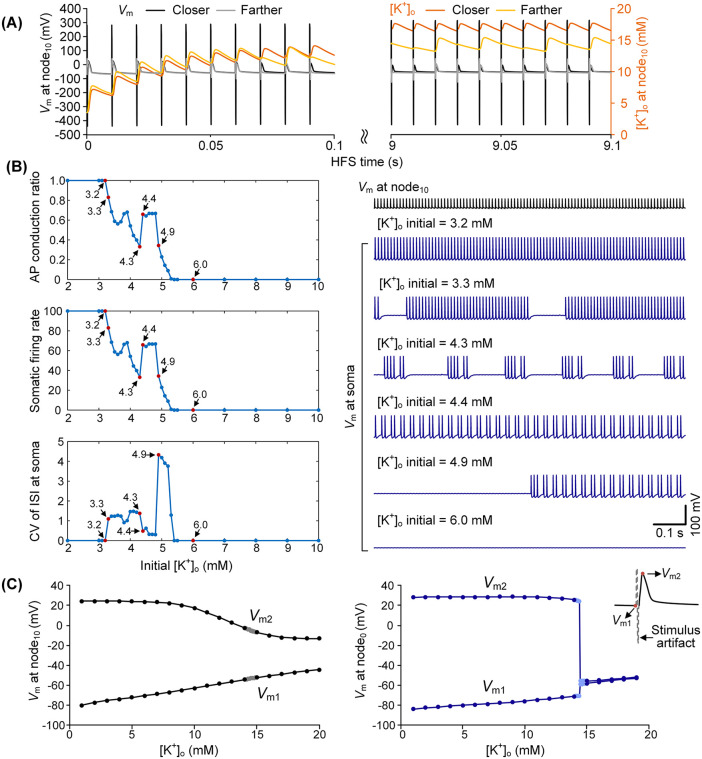
Effect of peri-axonal [K⁺]_o_ on neuronal firing patterns. (A) Membrane potentials and peri-axonal [K⁺]_o_ at node_10_ and its neighboring JXP during early and late HFS. Black/orange traces: neuron at (*x* = 30 µm, *z* = 30 µm); gray/yellow traces: neuron at (*x* = 60 µm, *z* = 60 µm). (B) Influence of initial [K⁺]_o_ on somatic firing patterns. (C) Mean resting membrane potential (*V*_m1_) and peak potential (*V*_m2_) as functions of [K⁺]_o_ at node_10_ (*left*) and node_0_ (*right*) in the absence of K⁺ accumulation. Gray and light blue dots indicate 0.1 mM subdivisions within the 14–15 mM range. Note the abrupt *V*_m1_ and *V*_m2_ changes at node_0_ between 14.4 and 14.5 mM.

We have previously reported that fluctuations in peri-axonal [K⁺]_o_ can induce intermittent axonal block thereby modulating firing activity [20]. In the present simulations, sustained HFS produced progressive K⁺ accumulation in the restricted peri-axonal space, with the rate and magnitude of elevation varying by spatial configuration (Fig 5A). This suggested that [K⁺]_o_ may serve as a critical intrinsic determinant of neuronal responses. To test this hypothesis directly, we systematically varied the initial [K⁺]_o_ and examined its impact on firing patterns.

The neuron was fixed at (*x* = 30 µm, *z* = 30 µm) with the SE positioned above node_10_, where each stimulus reliably initiated an AP (initiation ratio = 1.0; Fig 5B). Under this configuration, firing patterns were determined primarily by AP conduction. At low initial [K⁺]_o_ (≤3.2 mM), all APs propagated reliably to the soma, producing tonic, stimulus-locked firing with ISI CV values near zero (Fig 5B). A slight increase to ~3.3 mM induced conduction failures without affecting initiation, causing a bifurcation from tonic to clustered firing. Within the narrow range of ~3.3–4.3 mM, neurons consistently exhibited clustered firing, with cluster duration and compactness varying while overall ISI variability remained relatively stable. A further small increase (~4.3–4.4 mM) induced a bifurcation from clustered to regular firing, reflected by a doubling of somatic firing rate and a marked reduction in ISI variability. Beyond this point, firing again destabilized: at ~4.9 mM neurons reverted to long-cluster firing, and when [K⁺]_o_ exceeded ~5.3 mM, complete conduction block occurred, with no APs reaching the soma (Fig 5B).

To verify that these effects arose directly from potassium dynamics, we repeated simulations with K⁺ accumulation disabled and fixed [K⁺]_o_ levels imposed. Under these conditions, APs were highly reproducible across trials. Analysis of membrane potentials revealed that increasing [K⁺]_o_ gradually depolarized the resting potential (*V*_m1_), while AP peak potential (*V*_m2_) remained stable until critical thresholds were exceeded. At node_10_, *V*_m1_ progressively depolarized as [K⁺]_o_ rose, with *V*_m2_ remaining stable until [K⁺]_o_ > 10 mM, after which *V*_m2_ also declined, yielding progressively smaller APs (Fig 5C, *left*). At node_0_, a sudden decline in AP amplitude occurred between 14.4 and 14.5 mM, marking a bifurcation from tonic firing to complete block (Fig 5C, *right*). Such abrupt changes in excitability in response to minimal [K⁺]_o_ shifts are characteristic of bifurcation points in nonlinear systems.

Together, these results demonstrate that peri-axonal [K⁺]_o_ exerts strong control over firing stability. Even small changes in concentration can drive bifurcation-like transitions among tonic, clustered, regular, and blocked patterns. These findings highlight potassium dynamics as a key regulator of axonal excitability and motivate a more detailed dynamical analysis.

### 3.4. Nonlinear potassium dynamics underlying firing patterns

To further probe the dynamical basis of the observed firing patterns, we analyzed membrane potential (*V*_*m*_)–[K⁺]_o_ trajectories during steady HFS (~5 s after onset). These analyses revealed that both the neuron–SE spatial relationship and peri-axonal [K⁺]_o_ alter the stability landscape of axonal excitability.

**Fig 6:**
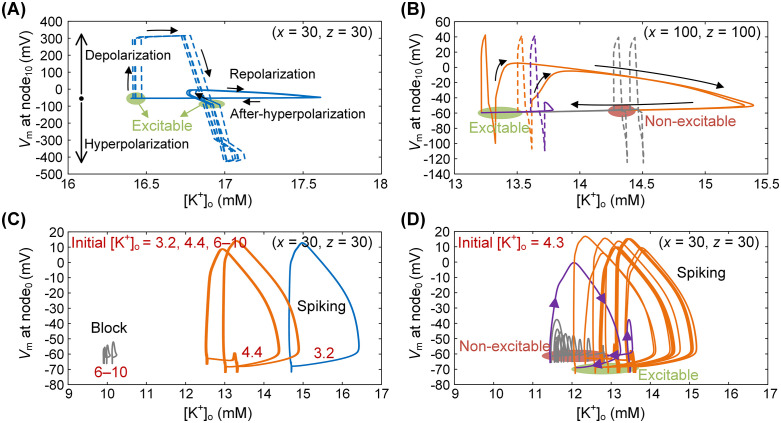
Nonlinear *V*_m_–[K⁺]_o_ dynamics underlying distinct firing patterns. (A, B) *V*_m_–[K⁺]_o_ trajectories at node_10_ showing continuous AP initiation in neurons near the SE (A) and intermittent initiation in neurons farther from the SE (B). (C, D) *V*_*m*_–[K⁺]_o_ trajectories at node_0_ showing tonic, intermittent, and blocked states under different initial [K⁺]_o_ (C), and clustered firing when [K⁺]_o_ = 4.3 mM (D). Corresponding firing activities for (C) and (D) are shown in Fig 5B. Blue, orange, and gray traces represent tonic, intermittent, and blocked firing, respectively. Green circles denote excitable states, red circles non-excitable states, and purple traces transitions between them.

For neurons positioned close to the SE (*x* = 30 µm, *z* = 30 µm), strong pulse-induced depolarization–hyperpolarization cycles produced modest [K⁺]_o_ elevations (~0.5 mM). The membrane then reliably returned to a stable excitable state (Fig 6A, green circle), allowing node_10_ to generate APs on every stimulus pulse and sustain tonic firing (Fig 6A). By contrast, distal neurons (*x* = 100 µm, *z* = 100 µm) failed to return immediately to the excitable resting state after each AP. Instead, they entered a transient non-excitable state corresponding to an unstable equilibrium, during which incoming pulses failed to evoke APs (Fig 6B, red circle). Only after several ineffective stimuli did the membrane gradually recover, restoring excitability (Fig 6B, green circle). This alternation between excitable and non-excitable states underlies the spatial dependence observed at node_10_, producing continuous AP initiation in neurons near the SE and intermittent initiation in those farther away.

Extracellular potassium exerted a similar modulatory effect. At low initial [K⁺]_o_ (3–5 mM), node_0_ remained in the spiking regime and supported faithful or intermittent firing (blue and orange traces in Fig 6C). At higher [K⁺]_o_ levels (6–10 mM), the membrane shifted into a persistent block state, abolishing spiking (gray traces in Fig 6C). At intermediate concentrations (~4 mM), the system exhibited bistability: trajectories alternated between excitable and blocked states along distinct paths, giving rise to clustered firing (Fig 6D).

These results reveal a strong nonlinear coupling between *V*_m_ and [K⁺]_o_. Small perturbations in extracellular potassium can drive the system across bifurcation thresholds, redirecting trajectories between stable and unstable equilibria. It is this nonlinear interplay, between external electric fields modulated by neuron–SE geometry and intrinsic ionic dynamics, that generates the diverse firing behaviors observed under uniform stimulation, including tonic, intermittent, clustered, and blocked states.

## 4. Discussion

This study demonstrates that neuronal firing patterns under HFS arise from the nonlinear interplay between external geometric factors and intrinsic ionic dynamics. To the best of our knowledge, this is the first work to provide a unified mechanistic account of the diverse neuronal responses observed during HFS, extending the classical conduction block framework into a nonlinear dynamical systems perspective. The mechanisms and implications are discussed below.

### 4.1. From conduction block to bifurcation mechanisms

Neural systems are inherently nonlinear, often exhibiting bifurcation, synchronization, and even chaotic dynamics [29–31]. A bifurcation occurs when small changes in control parameters trigger abrupt, qualitative changes in activity patterns [32,33]. Bifurcations have been implicated in a wide range of neural processes, from the onset of oscillations and seizures to transitions between spiking and resting states [32,34]. Within this framework, neuronal responses to HFS are best viewed not as linear input-output transformations, but as nonlinear state transitions modulated by the interaction between external stimulation and intrinsic ionic dynamics.

Traditionally, HFS has been interpreted through the conduction block hypothesis, in which high-frequency spiking elevates peri-axonal [K⁺]_o_, prolongs sodium-channel inactivation, and suppresses spike propagation [7,16,17,35,36]. While conduction block clearly occurs, this binary view cannot explain the reproducible diversity of firing patterns seen *in vivo*, where identical stimulation trains can produce tonic, clustered, intermittent, or fully blocked activity [37–40]. Our findings extend this framework by showing that conduction block represents only one extreme along a broader continuum of bifurcation-driven transitions.

Specifically, we found that conduction failures precede initiation failures as neuron–SE distance increases, producing sequential transitions from tonic to clustered and ultimately to low-rate regular firing (Figs 3–4). When initiation remained reliable but conduction failed intermittently, somatic firing became propagation-dependent, alternating between clustered and low-rate regular patterns (Fig 3A-3D; Fig 4A-4D). At greater distances, initiation itself became unreliable, leading to low-rate regular firing determined largely by initiation probability (Fig 3E and 4E). Importantly, spatial heterogeneity in extracellular fields contributed to this hierarchy [41]: closer neurons remained excitable even when [K⁺]_o_ rose to 15–20 mM, whereas farther neurons exposed to weaker electric fields were far more vulnerable, with conduction reliability strongly influenced by small potassium fluctuations (Fig 5A,5C; Fig 6A, 6B).

Across these transitions, peri-axonal [K⁺]_o_ acted as an intrinsic bifurcation parameter [42,43]. Fluctuations as small as a few tenths of a millimolar were sufficient to drive abrupt state changes, from tonic to clustered, clustered to regular, and ultimately to block (Figs 5 and 6). These transitions were not strictly sequential: depending on initial ionic conditions and membrane state, neurons could also switch bidirectionally between clustered and regular firing. This extreme sensitivity underscores how initial ionic states critically influence system trajectories and how even minor perturbations can reorganize firing in qualitatively distinct ways (Fig 6C and 6D).

Together, these findings indicate that axonal responses to HFS are best understood as bifurcation-driven switching of firing patterns, jointly governed by electrode-neuron geometry and potassium dynamics. Small spatial or ionic perturbations shift local fields and membrane excitability, pushing the system across bifurcation thresholds that restructure firing stability. Phase-space trajectories in the *V*_*m*_–[K⁺]_o_ plane further illustrate these transitions between stable and unstable equilibria, providing a compact dynamical explanation for the structured diversity of firing behaviors.

By reframing axons as nonlinear computational elements rather than passive conduits, this bifurcation framework bridges experimental observations with dynamical systems theory, offering a unified account of how HFS generates tonic, clustered, intermittent, or blocked activity under fixed stimulation parameters.

### 4.2 Implications for neurostimulation strategies

The ability of HFS to generate distinct firing patterns through nonlinear bifurcation mechanisms has direct engineering and physiological implications. Clustered firing, marked by alternating periods of activity and silence, can mimic burst signaling involved in synaptic plasticity and memory, with therapeutic benefits or potential cognitive side effects depending on the target circuit [44,45]. Tonic and intermittent regular firing may contribute to the suppression of pathological rhythms [7,9,40,46], whereas complete block produces lesion-like silencing, effectively suppressing pathological activity but also diminishing normal physiological signaling [5,7]. Which pattern prevails depends not only on stimulation frequency and amplitude but also on SE-neuron geometry and local ionic conditions.

Small variations in electrode placement or extracellular milieu may determine whether axons operate in tonic, clustered, or blocked regimes, thereby shaping both therapeutic efficacy and side-effect profiles. Several practical strategies emerge. First, geometric optimization, through micro-adjustments in lead depth or contact selection, can steer local fields toward parameter zones suited for desired modulation. Second, tuning pulse parameters (e.g., amplitude, width, or interphase structure) can shift trajectories away from unstable states and bias them toward stable firing regimes. Finally, closed-loop control incorporating extracellular potassium measurements or surrogate biomarkers could dynamically guide neuronal populations into target firing patterns. Together, these strategies may enhance the reliability and precision of neuromodulation therapies [3,47].

### 4.3 Limitations and future directions

This study has several limitations. First, although the model incorporated detailed axonal morphology and potassium dynamics, it did not capture the full heterogeneity of hippocampal neurons. Additional model parameters may also give rise to similar bifurcations in neuronal firing behavior. Moreover, features such as axonal branching were omitted and should be addressed in future work [37,48,49]. Second, the *in vivo* data were limited to somatic recordings, which provide only indirect measures of axonal conduction. Direct axonal or optogenetic voltage recordings will be essential to validate the predicted conduction failures. Third, potassium clearance was modeled using simplified diffusion and Na–K pump mechanisms, without accounting for astrocytic buffering or extracellular space constraints, both of which could substantially influence [K⁺]_o_ dynamics [16,50,51]. Finally, this work focused on acute stimulation, whereas clinical neuromodulation typically involves chronic and adaptive paradigms that likely engage long-term ionic and synaptic plasticity [3,5]. Extending the bifurcation framework to network-level models and chronic preparations will be critical for establishing its generality and translational relevance. Importantly, these limitations highlight clear avenues for future work without undermining the central conclusion: that neuron–SE geometry and potassium dynamics jointly modulate firing patterns through nonlinear bifurcations.

## Conclusion

In summary, this study establishes a bifurcation framework in which neuron–SE geometry and peri-axonal potassium dynamics serve as coupled but independently tunable parameters governing axonal excitability during HFS. This framework unifies diverse firing patterns, including tonic, clustered, intermittent, and blocked, within a single nonlinear dynamical perspective. In addition to extending mechanistic understanding beyond the binary conduction-block hypothesis, this work provides a principled design framework for selecting stimulation targets, optimizing electrode placement, and tuning stimulation pulses with predictable effects on firing distributions across axonal populations. By bridging biophysical mechanisms with translational strategies, this work lays the foundation for more reliable and precise neuromodulation therapies.

## Supporting information

S1 File Finite element modeling of the extracellular electric field. (DOCX)
