# Supplementary material for: Bifurcation of neural firing patterns driven by potassium dynamics and neuron–electrode geometry during high-frequency stimulation
Source: PLoS Comput Biol. 2026 Apr 28;22(4):e1014228. doi: 10.1371/journal.pcbi.1014228 (PMC13124058; doi:10.1371/journal.pcbi.1014228)
Supplement: S1 File — (DOCX) [file pcbi.1014228.s001.docx]

**Finite Element Modeling of the Extracellular Electric Field**

The extracellular electric field was computed using the finite element method implemented in COMSOL Multiphysics 5.3 (COMSOL Inc., Sweden) under the quasi-static approximation.

The stimulation electrode (SE) was modeled based on a concentric bipolar configuration (#CBCSG75, FHC Inc., USA). The inner pole, made of Pt/Ir (75 μm diameter), and the outer pole, made of a stainless steel tube (250 μm diameter), were both exposed over a length of 100 μm and separated by a 100 μm insulating epoxy junction. The inner pole was defined as the working electrode owing to its smaller surface area, while the outer pole served as the return electrode. During biphasic stimulation, the inner electrode alternated between cathodic (−0.3 mA) and anodic (+0.3 mA) current injection.

The extracellular space was modeled as a homogeneous and isotropic conductive medium with a conductivity of 0.286 S/m. The computational domain was defined as a rectangular volume of 100 μm × 2000 μm × 100 μm, which was sufficiently large to minimize boundary effects within the region of interest. The model was discretized using tetrahedral elements, with local mesh refinement applied near the electrode to ensure accurate resolution of the electric field. The electric potential was solved, and the extracellular electric field was computed as the negative gradient of the potential. The resulting potential distribution was sampled at multiple spatial locations and mapped onto the neuronal model according to compartment positions.
